# Correction to “Prognostic Implications of Heart Rate Score and Its Temporal Change in Left Ventricular Systolic Dysfunction: Insights From the HINODE Study”

**DOI:** 10.1002/joa3.70306

**Published:** 2026-02-26

Okada M, Inoue K, Tanaka N, et al. “Prognostic Implications of Heart Rate Score and Its Temporal Change in Left Ventricular Systolic Dysfunction: Insights From the HINODE Study,” *Journal of Arrhythmia* 2025 41 no. 6: e70216. https://doi.org/10.1002/joa3.70216.

In Figure 4, the marker and confidence interval bar for the subgroup “LRL < 50 bpm” were plotted incorrectly. The numeric values reported in the text are correct. The corrected Figure 4 is provided below. This correction does not affect the results or conclusions.

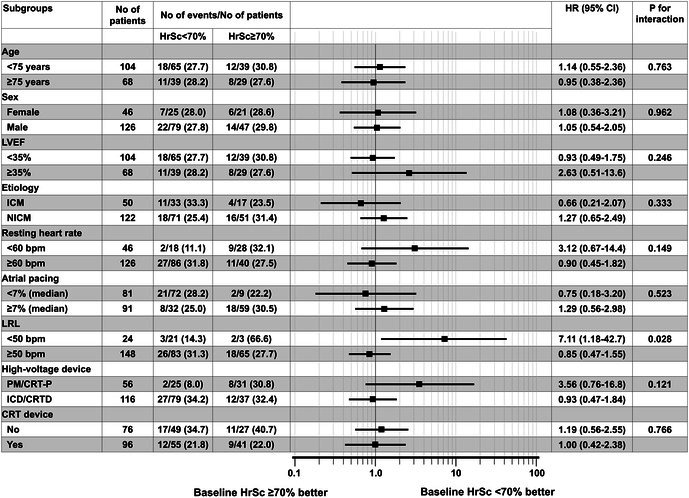

We apologize for this error and any confusion it may have caused.

